# Reversed predator–prey cycles are driven by the amplitude of prey oscillations

**DOI:** 10.1002/ece3.4184

**Published:** 2018-05-24

**Authors:** Ellen van Velzen, Ursula Gaedke

**Affiliations:** ^1^ Department of Ecology and Ecosystem Modelling Institute of Biochemistry and Biology University of Potsdam Potsdam Germany

**Keywords:** coevolution, ecoevolutionary dynamics, predator–prey dynamics, top‐down control

## Abstract

Ecoevolutionary feedbacks in predator–prey systems have been shown to qualitatively alter predator–prey dynamics. As a striking example, defense–offense coevolution can reverse predator–prey cycles, so predator peaks precede prey peaks rather than vice versa. However, this has only rarely been shown in either model studies or empirical systems. Here, we investigate whether this rarity is a fundamental feature of reversed cycles by exploring under which conditions they should be found. For this, we first identify potential conditions and parameter ranges most likely to result in reversed cycles by developing a new measure, the *effective prey biomass*, which combines prey biomass with prey and predator traits, and represents the prey biomass as perceived by the predator. We show that predator dynamics always follow the dynamics of the effective prey biomass with a classic ¼‐phase lag. From this key insight, it follows that in reversed cycles (i.e., ¾‐lag), the dynamics of the actual and the effective prey biomass must be in antiphase with each other, that is, the effective prey biomass must be highest when actual prey biomass is lowest, and vice versa. Based on this, we predict that reversed cycles should be found mainly when oscillations in actual prey biomass are small and thus have limited impact on the dynamics of the effective prey biomass, which are mainly driven by trait changes. We then confirm this prediction using numerical simulations of a coevolutionary predator–prey system, varying the amplitude of the oscillations in prey biomass: Reversed cycles are consistently associated with regions of parameter space leading to small‐amplitude prey oscillations, offering a specific and highly testable prediction for conditions under which reversed cycles should occur in natural systems.

## 1 INTRODUCTION

It is now well‐established that evolutionary changes can be rapid and may occur on the same timescales as ecological changes, such as changes in population densities (DeLong et al., 2016; Ellner, Geber, & Hairston, 2011; Hairston, Ellner, Geber, Yoshida, & Fox, 2005). When such rapid evolutionary changes occur in traits that are ecologically relevant, such as traits involved in resource acquisition or interspecies interactions, the mutual feedback between ecological and evolutionary processes can give rise to ecoevolutionary dynamics (Post & Palkovacs, 2009; Schoener, 2011).

A striking number of such rapid trait changes have been found in traits governing predator–prey interactions, ranging from the evolution of prey defenses against predators (Becks, Ellner, Jones, & Hairston, 2010; Frickel, Sieber, & Becks, 2016; Jones et al., 2009; Wei, Kirby, & Levin, 2011; Yoshida, Jones, Ellner, Fussmann, & Hairston, 2003; Yoshida et al., 2007) to the evolution of increased prey capture or countermeasures against prey defense in predators (Frickel et al., 2016; Grant & Grant, 2002; Hairston et al., 1999; Hall, Scanlan, & Buckling, 2011). When such traits evolve on the same timescale as predator–prey population dynamics, the resulting ecoevolutionary feedbacks can qualitatively change the shape and timing of predator–prey cycles. Predator–prey systems without evolution exhibit “classic” ¼‐lag cycles, where predator dynamics follow those of the prey with a lag of a quarter of the cycle period (Bulmer, 1975; Rosenzweig & MacArthur, 1963). However, when the level of prey defense can rapidly evolve in response to changing predator densities, predator and prey oscillations may show antiphase dynamics instead (Becks et al., 2010; Cortez, 2011; Cortez & Ellner, 2010; Yoshida et al., 2003). This result has been consistently shown in theoretical and experimental studies, and consequently, antiphase predator–prey cycles have become known as a “smoking gun” for the presence of rapid prey evolution (Hiltunen, Hairston, Hooker, Jones, & Ellner, 2014).

When prey and predators can both evolve rapidly, antiphase cycles may be found as well (Frickel et al., 2016; Mougi & Iwasa, 2011), but coevolution may also result in different cycle types that are not found under prey evolution alone. Strikingly, coevolution can result in reversed predator–prey cycles: predator peaks preceding prey peaks, rather than vice versa (Cortez, 2015; Cortez & Weitz, 2014). Such dynamics have been empirically demonstrated as well (Wei et al., 2011), but only rarely, and in model studies, they have also been demonstrated only under restrictive conditions: First, trait changes must be very rapid, even more rapid than ecological changes; second, selection on prey and predator traits must be disruptive, resulting in sudden shifts between extreme phenotypes on both trophic levels (highly edible to highly inedible prey, inoffensive to highly offensive predators); and third, costs for predator offense must be high (Cortez, 2015; Cortez & Weitz, 2014). This raises two questions: First, how common should we expect reversed cycles to be in empirical systems? And second, can we narrow down under which conditions, if any, they are most likely to occur?

### 1.1 Predicting reversed cycles: the effective prey biomass

In this section, we will first predict which general conditions are most likely to yield reversed cycles, using a new concept we have called the *effective prey biomass* (van Velzen & Gaedke, 2017). This is a measure of prey biomass as perceived by the predator, reflecting the fact that not all prey biomass present can be captured and assimilated by the predator. For example, in an experimental rotifer‐algal system with two prey clones (i.e., completely edible and completely inedible algae; Becks et al., 2010; Becks, Ellner, Jones, & Hairston, 2012), the inedible clone is unavailable to the predator. From the predator’s point of view, the inedible clone might as well not be present: The effective prey biomass consists only of the biomass of the edible clone. The phase lag between the dynamics of the predator and the edible prey clone is a classic ¼‐lag, both in predictions from a mathematical model and in the actual experimental dynamics (Becks et al., 2012; Jones & Ellner, 2007). Thus, while the dynamics between predator and total prey biomass are antiphase (Becks et al., 2010, 2012), the phase lag between predator and the *effective* (edible) prey biomass remains a ¼‐lag.

We extend the above insight here as follows: In an evolutionary or coevolutionary predator–prey system, predator dynamics are regulated by the *effective prey biomass* in the same way that they are regulated by the total prey biomass in a nonevolutionary model. This means that predator dynamics are expected to follow the *effective prey* dynamics with a ¼‐lag. We define the effective prey biomass here as the total prey biomass, multiplied by the *net gain to the predator per unit prey biomass present*. This net gain can be reduced (or increased) by changes in prey or predator traits in various ways, which can be divided into two distinct categories:

1. *Precapture effects:* Most straightforwardly, part of the actual prey biomass may not be available to be captured by the predator, for example, because they are too large to be ingested (Becks et al., 2010, 2012). The degree of vulnerability to the predator has been called *edibility* (Becks et al., 2010; Jones & Ellner, 2007), and we keep this terminology here. The edibility can be influenced by any form of defense allowing the prey to avoid being captured by the predator. Examples include formation of colonies too large for predators to ingest (Becks et al., 2010), biofilm formation in bacteria to avoid grazing by suspension‐feeding protozoans (Matz & Kjelleberg, 2005), or resistance against viral infections (Frickel et al., 2016). The edibility is also influenced by predator’s countermeasures against defense, such as a predator’s increase in gape size to capture larger prey (Kopp & Tollrian, 2003) or a parasite’s increased virulence (Frickel et al., 2016; Hall et al., 2011).
2. *Postcapture effects:* The gain to the predator per captured prey may be lowered. As the most straightforward example, prey defense may take the form of avoiding digestion rather than avoiding capture (Meyer, Ellner, Hairston, Jones, & Yoshida, 2006; Porter, 1973). Less intuitively, it can also be affected by the predators’ offense traits. If a high‐offense strategy is energetically costly, such as increased swimming speed, the conversion efficiency of captured prey into predator biomass is reduced (Kiørboe, 2011; Pahlow & Prowe, 2010). Similarly, a lower conversion efficiency can result from increased body size as an offensive strategy (Kopp & Tollrian, 2003) when this requires more captured prey to maintain. At last, an increase in offense may come at the cost of increased mortality, for example, when increased swimming speed of the predator results in encounter rates with both prey and with natural enemies (Kiørboe, 2011). In all these cases, the cost incurred from the high‐offense strategy needs to be taken into account when determining the effective prey biomass. This postcapture profitability of the prey to the predator thus may consist of various processes, which from here on we will together refer to as the prey’s *conversion efficiency*.

Thus, the effective prey biomass can be defined as a combination of three elements: first, the total prey biomass (we will call this the *actual prey biomass* from here on when contrasting it with the actual prey biomass); second, the preattack profitability or *edibility*; and third, the postattack profitability or *conversion efficiency*. It should be noted here that in any predator–prey interaction, there are obviously always many factors influencing the edibility of the prey, the ability of the predator to digest captured prey, or the level of energetic or mortality costs of searching for prey. What is important is not the presence of any such factors, but that when they change on the same timescale as the population dynamics due to contemporary evolution in the prey or the predators, this can result in deviations between the dynamics of the actual and the effective prey biomass. From this follows a straightforward but critical insight: The predator–prey phase lag is determined by the phase lag between actual and effective prey biomass. Thus, for example, antiphase predator–prey cycles result when the effective prey biomass is a ¼‐lag behind the actual prey biomass.

**Figure 1:**
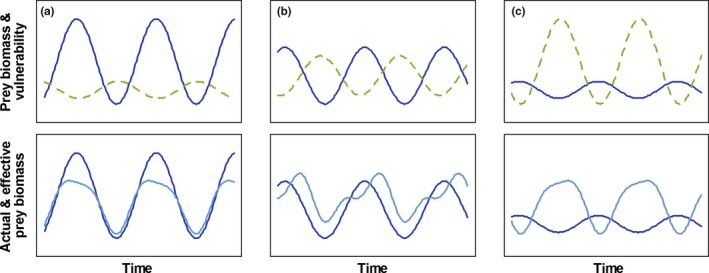
The effect of the amplitude of prey oscillations on the phase lag between actual and effective prey biomass. All three cases (a–c**)** show out‐of‐phase oscillations between actual prey biomass and edibility (top panels); the relative amplitudes of their oscillations determine the lag between actual and effective prey biomass. (a) Oscillations in actual prey biomass (solid blue line) are much stronger than those in edibility (top panel, dashed green line); as a result, dynamics of the effective prey biomass (bottom panel, light blue line) are in phase with those of the actual prey biomass. (b) Oscillations in actual prey biomass and edibility are of similar amplitude; the phase relationship between actual and effective prey biomass is around a ¼‐lag. (c) Oscillations in actual prey biomass are small, while oscillations in edibility are strong; dynamics of the effective prey biomass are mostly determined by the dynamics of the edibility, and actual and effective prey biomass are in antiphase with each other

We use the above approach to derive a general condition for reversed predator–prey cycles. In this dynamic, prey dynamics follow behind predator dynamics with a ¼‐lag; stated differently, predator dynamics follow prey dynamics with a ¾‐lag. This means that the dynamics of actual and effective prey biomass must be in antiphase with each other (i.e., the lag between actual and effective prey biomass must be ½). In other words, the effective prey biomass must be high when the actual prey biomass is low, and vice versa. It directly follows that the two trait‐based elements of the effective prey biomass, edibility, and conversion efficiency must be high when the actual prey biomass is low and must be low when actual prey biomass is high. Moreover, the impact of changes in edibility and the conversion efficiency on the effective prey biomass must outweigh the impact of changes in actual prey biomass (see Figure 1). We therefore derive the following hypothesis: *reversed predator–prey cycles should be found when oscillations in actual prey biomass are of small amplitude, so that the dynamics of the effective biomass are driven mainly by trait dynamics*.

To test this hypothesis, we simulate the dynamics of a mathematical predator–prey model with coevolution, where we use two methods to vary the amplitude of prey oscillations. First, we systematically vary two parameters affecting the strength of top‐down control (predator mortality and the efficiency of defense). Under strong top‐down control, predator biomass is high and suppresses prey peaks, resulting in smaller‐amplitude oscillations in prey biomass. Our hypothesis predicts that reversed cycles should be found in the regions of parameter space where top‐down control is severe. Second, we model prey growth using two functions in which the costs of defense are differently expressed: In the first model (standard logistic growth), costs become less severe with increasing prey biomass, while in the second model (prey growth with self‐limitation), costs are independent of prey biomass. The first model should result in more pronounced prey oscillations and thus should be less likely to yield reversed cycles than the second model.

Our simulation results confirm all our predictions. We show that the phase lag between predator biomass and effective prey biomass is always a classic ¼‐lag, confirming the validity of the conceptual framework we developed. We further confirm that reversed cycles are consistently associated with small‐amplitude prey oscillations and are strongly promoted by severe top‐down control. The close correspondence between our predictions and our results strongly supports the effective prey biomass as a useful new tool for understanding and predicting predator–prey dynamics under coevolution.

## 2 METHODS

### 2.1 Model structure

Following previous work on predator–prey coevolution (Cortez & Weitz, 2014; Mougi, 2012; Mougi & Iwasa, 2011; Tien & Ellner, 2012), we model a single prey (*N*) and predator (*P*), each with an adaptive trait (defense *u* and offense *v*, respectively). The two traits together determine the capture rate of the predator. The ecological predator–prey dynamics are described as follows:

(1) $$\begin{array}{cc} \frac{dN}{dt} & \;=\;\left[f\left(N,u\right)\;-\;g\left(N,P,u,v\right)\right]N\\ \frac{dP}{dt} & \;=\;\left[\gamma \left(N,u,v\right)\;-\;d\right]P \end{array}$$

here, *f* denotes prey growth, *g* the per capita predation rate, and γ a combination of the per capita predation rate with the conversion of captured prey into predator biomass (i.e., predator growth). *d* is the per capita mortality rate of the predator.

For *f*(*N*,*u*), we compare and contrast two similar models for density‐dependent prey growth:

(2) $$\begin{array}{cc} f_{1}\left(N,u\right) & \;=\;r\left(u\right)\left(1-\frac{N}{K}\right)\;\left(\text{model}\;1\right)\\ f_{2}\left(N,u\right) & \;=\;r\left(u\right)-\frac{N}{K_{r}}\;\left(\text{model}\;2\right) \end{array}$$

Model 1 is the standard expression for logistic growth, with intrinsic growth rate *r* and carrying capacity *K*. Model 2 is a common alternative expression of self‐limitation in growth (Abrams, 2000a; Abrams & Cortez, 2015; Mougi & Iwasa, 2011) where *K*_*r*_ does not denote the carrying capacity, but is an inverse of the mortality rate and represents the severity of self‐limitation; the carrying capacity is instead *r*(*u*)*K*_*r*_. The major difference between the two models is in how they affect the costs of defense. In both models, we assume a trade‐off between defense *u* and the intrinsic growth rate *r*:

(3) $$r\left(u\right)\;=\;r_{0}e^{-c_{N}u^{2}}$$

where *r*_0_ is the maximum growth rate, attained when *u *=* *0, and *c*_*N*_ denotes the costliness of defense. In this study, we assume *r*_0_ = 1 (Table 1); this parameter choice means that the two models are identical without evolution and with completely undefended prey (*u *=* *0). However, the severity of the costs is not solely determined by the reduction in *r*. In Model 1, the impact of *r* on the dynamics decreases as prey biomass approaches the carrying capacity, leading to a reduction in the severity of the costs at high prey biomass (and in the extreme case when prey biomass is at the capacity, costs are not impacting the dynamics at all). Thus, in Model 1, the same level of defense comes at a higher cost when prey biomass is low, and at a lower cost when prey biomass is high. Conversely, in Model 2, the severity of the costs is independent of prey biomass. Such differences in cost dynamics have been shown to impact predator–prey dynamics: Tien and Ellner (2012) revealed that when costs increase with prey biomass, dynamics are stabilized more strongly than when costs are independent of prey biomass. The reverse should be found when costs decrease with prey biomass: Model 1 should generate more pronounced oscillations in prey biomass than model 2. Thus, we expect that Model 2 is more likely to result in reversed predator–prey cycles.

**Table 1:** List of parameters and parameter values

Parameters	Description	Value
*K*,* K*_*r*_	Carrying capacity of prey	2.5
*r*_0_	Maximum growth rate prey	1.0
ε_0_	Maximum conversion efficiency	0.5
**θ**	**Efficiency of defense**	**5–12.5**
***d***	**Per capita mortality predator**	**0.005–0.075**
*a*_0_	Maximum attack rate	1.0
*h*	Handling time	0.1
*c*_*N*_	Costliness of defense	4.0
*c*_*P*_	Costliness of offense	3.5
*G*_*N*_	Speed of adaptation prey	0.01
*G*_*P*_	Speed of adaptation predator	0.01

Marked in bold are the parameters we varied for our analysis.

Predation is modeled as a Holling type II functional response with attack rate *a*(*u*,*v*) and handling time *h*:

(4) $$g\left(N,P,u,v\right)\;=\;\frac{a\left(u,v\right)P}{1+ha\left(u,v\right)N}$$

The levels of defense and offense together determine the vulnerability of the prey to the predator, given by the attack rate *a*(*u, v*). This is modeled as a sigmoidal function of the difference between the two trait values (*u*–*v*), which assumes that the capture rate decreases with increasing defense and increases with increasing offense (Mougi & Iwasa, 2011; van Velzen & Gaedke, 2017):

(5) $$a\left(u,v\right)\;=\;\frac{a_{0}}{1+e^{\theta \left(u-v\right)}}$$

here, *a*_0_ is the maximum attack rate, achieved if *v* ≫ *u* (high offense and low defense). Conversely, if defense is very high compared with offense (*u* ≫ *v*), the attack rate approaches zero. θ determines the sensitivity of *a* to the difference between the trait values; higher values for θ can thus be interpreted as a higher effectiveness of defense. The function in Equation (5) represents what has been called a unidirectional trait axis (Abrams, 2000b), a common assumption in models of coevolution that is applicable to many predator–prey interactions (Nuismer, Ridenhour, & Oswald, 2007).

Predator growth γ is calculated by multiplying the per capita predation rate with the conversion efficiency of prey into predator biomass:

(6) $$\gamma \left(N,u,v\right)\;=\;\varepsilon \left(v\right)\frac{a\left(u,v\right)N}{1+ha\left(u,v\right)N}$$

We assume here a trade‐off between offense and the conversion efficiency ε (Kiørboe, 2011; Mougi, 2012):

(7) $$\varepsilon \left(v\right)\;=\;\varepsilon_{0}e^{-c_{P}v^{2}}$$

where ε_0_ is the maximum conversion efficiency at *v *=* *0, and *c*_*P*_ represents the costliness of offense.

At last, the evolutionary dynamics of *u* and *v* are modeled using the adaptive dynamics approach (Abrams, 2001). The speed and direction of evolutionary change are proportional to the fitness gradient for frequency‐independent selection, evaluated at the current trait value:

(8) $$\begin{array}{cc} & \frac{du}{dt}\;=\;G_{N}\frac{\partial }{\partial u}\left[\frac{1}{N}\frac{dN}{dt}\right]e^{-\alpha /u}\\ & \frac{dv}{dt}\;=\;G_{P}\frac{\partial }{\partial v}\left[\frac{1}{P}\frac{dP}{dt}\right]e^{-\alpha /v} \end{array}$$

*G*_*N*_ and *G*_*P*_ represent the additive genetic variation in the prey and predator populations, determining the speed of evolutionary change relative to the ecological dynamics. We assume here that the speed of evolutionary change is relatively slow compared with the ecological dynamics (*G*_*N*_ = *G*_*P*_ **= 0.01), representing gradual shifts in trait values. The exponential functions in Equation (8) are boundary functions (Abrams & Matsuda, 1997) restricting the dynamics of *u* and *v* to positive values by decreasing the speed of evolutionary change when *u* or *v* very closely approach zero (α* *= 0.001).

### 2.2 Effective prey biomass

Following the guidelines we outlined in the Introduction, we define the effective prey biomass as the amount of prey biomass that can be captured by the predator and converted into predator biomass: If prey is abundant, but the predator is physically incapable of capturing or digesting them, the effective prey biomass is zero. In the model as defined in Equations(1), (2), (3), (4), (5), (6), (7) above, the effective prey biomass is affected by preattack effects (*edibility*), when evolution of defense and/or offense changes the attack rate *a*, as well as by postattack effects (*conversion efficiency*) through reduction of the predator–prey conversion efficiency ε as a cost of high offense.

As the effective prey biomass *N*_eff_ must be measured in the same units as the actual prey biomass *N*, the definitions of the edibility and digestibility should both be dimensionless numbers (e.g., fractions). We therefore calculate *N*_eff_ as a combination of prey biomass *N*, the predator’s attack rate *a* relative to the maximum attack rate *a*_0_, and the predator’s conversion efficiency ε relative to the maximum conversion efficiency ε_0_:

(9) $$N_{\mathrm{eff}}\;=\;N\times \frac{a\left(u,v\right)}{a_{0}}\times \frac{\varepsilon \left(v\right)}{\varepsilon_{0}}$$

### 2.3 Simulations

We systematically varied two parameters that affect the level of top‐down control in the system: the predator mortality rate *d*, and the effectiveness of defense θ. These two parameters were varied on a 25 × 25 grid, with 0.005 ≤ *d *≤* *0.075 and 5 ≤ θ* *≤ 12.5. All other parameter values we used are provided in Table 1. Numerical simulations were run in Wolfram Mathematica 10 for 50,000 time steps; all calculations were based on the 20,000 time steps. The simulated time series used for the calculations were all generated with a step size of 1.

### 2.4 Phase relationships

The phase lags φ between predator and actual prey, between predator and effective prey, and between effective and actual prey were calculated using the dominant frequency of the Fourier transform of the last 20,000 time steps of the simulated time series (Bulmer, 1975; Mougi, 2012; Mougi & Iwasa, 2011; Platt & Denman, 1975) using Mathematica’s Fast Fourier Transform function. Phase lags are expressed as 0 ≤ φ* *≤ 1, where 0 and 1 indicate in phase cycles (no lag). φ* *≈ 0.25 indicates classic ¼‐lag cycles, and φ* *≈ 0.5 indicates antiphase cycles. A predator–prey phase lag longer than antiphase (φ* *> 0.5) denotes a reversal in the peaks of prey and predator abundance, with “true” reversed cycles (i.e., peaks in prey biomass following those of the predator with a ¼‐lag) at φ* *≈ 0.75.

### 2.5 Amplitude of oscillations

As a measure of the amplitudes, we calculated the standard deviations of the dynamics of the actual prey biomass σ_*N*_, and of the product of edibility and conversion efficiency σ_*a*ε_. To represent the magnitude of oscillations in actual prey biomass relative to those in edibility and conversion efficiency, we then calculated the relative variability over time *V*_rel_ as follows:

(10) $$V_{\mathrm{rel}}\;=\;\frac{\sigma_{N}}{\sigma_{N}+\sigma_{a\varepsilon }}$$

This results in 0 ≤ *V*_rel_ ≤ 1. *V*_rel_ > 0.5 indicates that oscillations in prey biomass are stronger than those in edibility and conversion efficiency; thus, the actual prey biomass has the strongest impact on the effective prey biomass. Conversely, *V*_rel_ < 0.5 indicates that the impact of edibility and conversion efficiency outweigh the impact of the actual prey biomass. According to our hypothesis, reversed cycles should be found when *V*_rel_ is small (*V*_rel_ ≪ 0.5).

## 3 RESULTS

### 3.1 Relative variability in prey biomass

**Figure 2:**
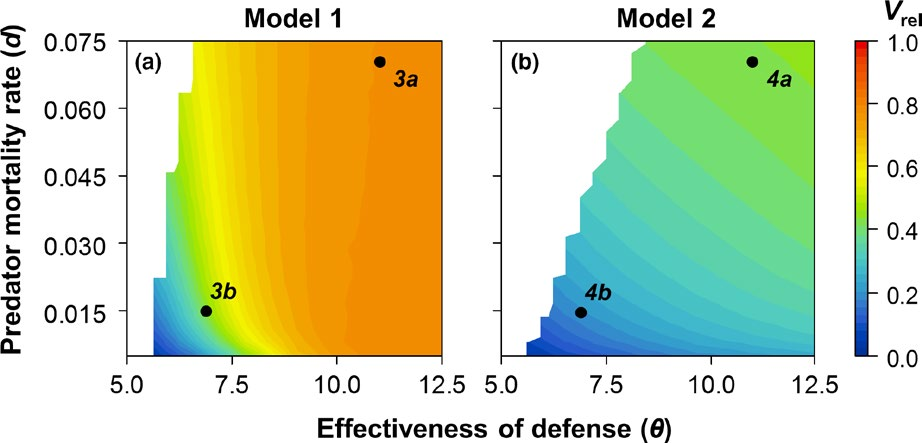
The relative variability in actual prey biomass *V*_*rel*_ as a function of predator mortality *d* and the effectiveness of defense θ. (a) Model 1; (b) Model 2. All other parameter values are given in Table 1. Colors denote the relative variability in prey biomass dynamics (see Section 2); white regions indicate stable equilibria. *3a,b* and *4a,b* refer to the parameter combinations for which the dynamics are shown in Figures 3 and 4

In order to relate the predator–prey phase lag to the relative variability in prey biomass *V*_rel_, we first confirm that the two methods we used for manipulating *V*_rel_ had the intended effect. Model 2 gives rise to smaller relative prey variability than Model 1 over the entire parameter space studied, with the exception of a narrow range where predator mortality and effectiveness of defense are both very low (Figure 2). These patterns were insensitive to the exact measure of variability used (Supporting information: Appendix A: Figure A1). This difference in relative variability is mainly driven by strong differences in prey variability σ_*N*_, with the variability in edibility and conversion efficiency σ_*a*ε_ being more similar across the two models (Supporting information: Appendix A: Figure A2).

In addition to the difference between the two models, the two parameters we varied both affected the relative variability in the expected direction: In Model 1, the effectiveness of defense clearly had the stronger effect (Figure 2a), while in Model 2, the two parameters were more equal in effect (Figure 2b). In both models, the smallest *V*_rel_ was consistently found when predator mortality and effectiveness of defense were both low (Figure 2).

### 3.2 Ecoevolutionary dynamics

Based on the results shown in Figure 2, we now compare the ecoevolutionary dynamics under weak top‐down control (high predator mortality *d* and high effectiveness of defense θ) and strong top‐down control (low *d* and low θ).

#### 3.2.1 Model 1 (promoting high‐amplitude prey oscillations)

**Figure 3:**
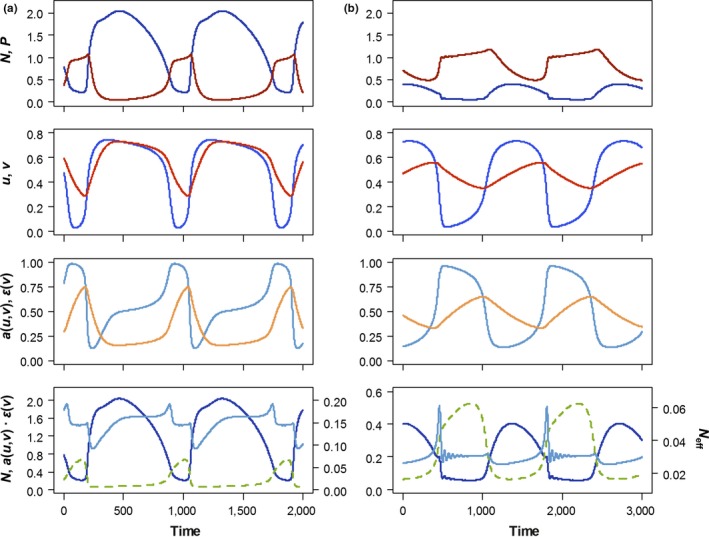
Ecoevolutionary dynamics of Model 1. (a) weak top‐down control: *d *=* *0.07, θ* *= 11; (b) strong top‐down control: *d *=* *0.015, θ* *= 7. Other parameter values are given in Table 1. Upper row: biomass dynamics of prey (blue) and predator (red). Second row: dynamics of defense (*u*, blue) and offense (*v*, red). Third row: edibility (attack rate relative to its maximum, *a*(*u*,*v*)/*a*_0_; light blue) and conversion efficiency (relative to its maximum, ε(*v*)/ε_0_; orange). Lower panels: effective prey biomass (solid light blue line), calculated by multiplying the actual prey biomass (solid dark blue line) with the product of edibility and conversion efficiency (dashed green line)

Maximum and minimum predator biomass are both lower under the weak top‐down control scenario (Figure 3a) than under the strong top‐down control scenario (Figure 3b). As a result, mean predator biomass is higher under strong top‐down control, peaks in prey biomass are suppressed, and oscillations in prey biomass are of smaller amplitude (Figure 3a,b, first row).

The interplay between biomass and trait dynamics follows a consistent pattern across both scenarios. Prey biomass *R* and defense *u* are strongly temporally synchronized, so that increases in prey biomass are associated with increases in defense, and decreases in prey biomass are associated with decreases in defense (Figure 3, first and second row). Offense evolves in response to defense, always lagging slightly behind: increasing to counter the prey’s defense when defense is high, and decreasing to reduce costs when defense is low (Figure 3, second row). As a result of these trait dynamics, edibility (standardized attack rate, *a*/*a*_0_) and conversion efficiency (ε/ε_0_) decrease as prey biomass increases (Figure 3, third row). Therefore, the product of edibility and conversion efficiency is low when prey biomass is high, and high when prey biomass is low (Figure 3, bottom row).

The dynamics of the effective prey biomass are determined by the combined dynamics of actual prey biomass, edibility, and conversion efficiency (Figure 3, bottom row). The effective prey biomass dynamics show deviations from those of the actual prey biomass at both weak and strong top‐down control: The effective prey biomass declines when the actual prey biomass increases, mainly driven by the sharp decline in edibility (Figure 3, third and bottom row), and the peak in the effective prey biomass is delayed with respect to the peak in the actual prey biomass (Figure 3, bottom row). These deviations are far more pronounced under strong top‐down control. When top‐down control is weak, oscillations in actual prey biomass far outweigh those in edibility and conversion efficiency (Figures 2a and 3a, bottom row); as a result, the effective prey biomass tends to be high when the actual prey biomass is high, and the delay is around a quarter of the cycle period (Figure 3a, bottom row). When top‐down control is strong, oscillations in actual prey biomass are smaller (Figure 2a), and the effective prey biomass is closer to being in antiphase with the actual prey biomass.

#### 3.2.2 Model 2 (promoting small‐amplitude prey oscillations)

**Figure 4:**
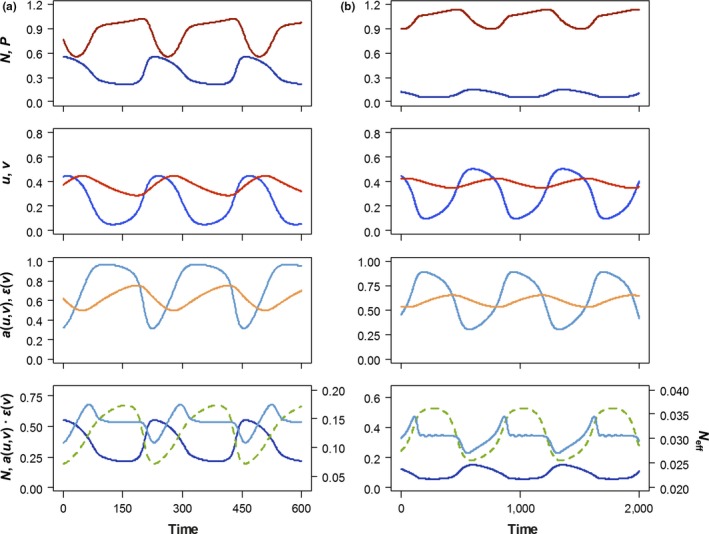
Ecoevolutionary dynamics of Model 2. (a) weak top‐down control: *d *=* *0.07, θ* *= 11; (b) strong top‐down control: *d *=* *0.015, θ* *= 7. Other parameter values are given in Table 1. Line types and colors have the same meaning as in Figure 3

The trade‐off structure of Model 2 yields prey oscillations of much smaller amplitude for the same parameter combinations than Model 1 (Figures 2a,b, 3, 4, first row); and again, stronger top‐down control results in smaller‐amplitude prey oscillations than weak top‐down control (Figure 4, first row). Despite these differences, the general ecoevolutionary dynamics of Model 2 are very similar to those of Model 1. We again find temporal synchronization between prey biomass and trait dynamics, so that prey become less edible and less digestible as they become more abundant, and vice versa (Figure 4, second and third row).

The similarities in ecoevolutionary dynamics, combined with the smaller‐amplitude oscillations in actual prey biomass, result in effective prey biomass dynamics that are more strongly determined by edibility and conversion efficiency (Figure 4, bottom row). When top‐down control is strong, actual and effective prey biomass are now even closer to being in antiphase with one another (Figure 4b, bottom row).

### 3.3 Phase relationships

#### 3.3.1 Predator–effective prey

**Figure 5:**
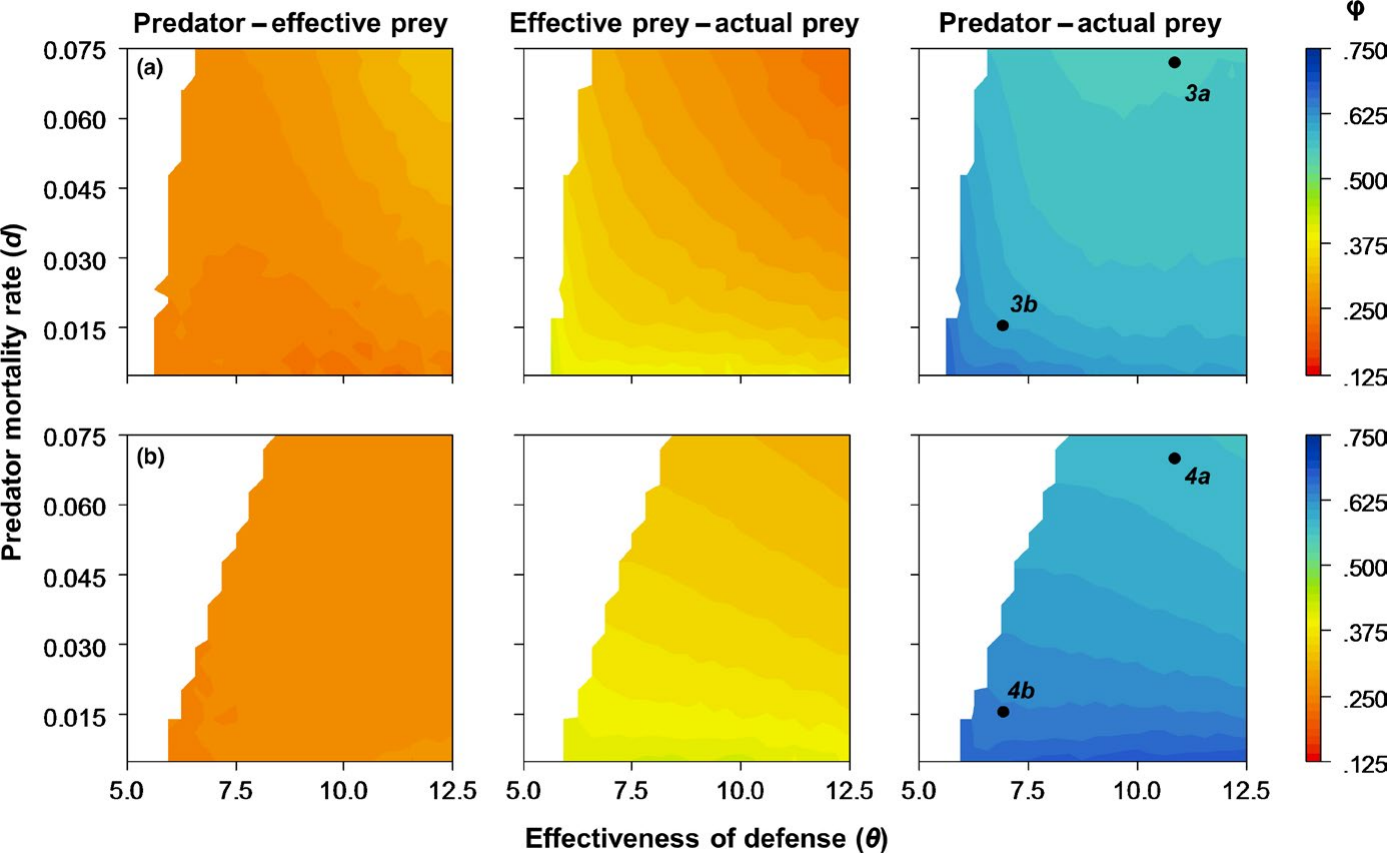
Phase lags between predator biomass and effective prey biomass (left), between effective and actual prey biomass (middle) and between predator and actual prey biomass (right), when varying predator mortality *d* and the effectiveness of defense θ. Colors denote the phase lag, as given in the legend on the right; white regions indicate stable equilibria. (a) Model 1; (b) Model 2. Parameter values are given in Table 1. *3a,b* and *4a,b* refer to the parameter combinations for which the dynamics are shown in Figures 3 and 4

Confirming the central concept underlying our approach, predator biomass dynamics follow those of the effective prey biomass with a ¼‐lag across both models and for the entire parameter space studied (Figure 5, left column).

#### 3.3.2 Actual prey–effective prey

Because the increase in actual prey biomass is always associated with a decrease in effective prey biomass, the effective prey biomass always lags behind the actual prey biomass (Figure 5, middle column; Figures 3, 4, bottom row). This lag is shortest at high predator mortality and high effectiveness of defense; moreover, it is shorter in Model 1 than in Model 2, reflecting the relatively close match between actual and effective prey biomass in Model 1 when top‐down control is weak (Figure 3a). Lower effectiveness of defense and lower predator mortality both increase the lag, and in both models, the longest lags are found where oscillations in actual prey biomass are smallest compared with those of edibility and conversion efficiency (Figure 2). Thus, in Model 1, the longest lags occur when predator mortality and effectiveness of defense are both low (Figure 5a); in Model 2, the longest lags occur when predator mortality is low, with the effectiveness of defense having a smaller effect (Figure 5b).

#### 3.3.3 Predator–prey

At last, the predator–prey phase lag is the sum of the ¼‐lag between predator and effective prey and the lag between actual prey and effective prey. Thus, the longest phase lags are found under low predator mortality and low effectiveness of defense in Model 1 (Figure 5a, right column), and under low predator mortality in Model 2 (Figure 5b, right column). For the parameter space studied, we always find predator–prey phase lags longer than antiphase. The dynamics of both models indeed always show predator peaks preceding prey peaks, rather than vice versa (Figures 3, 4). Phase lags are longer in Model 2 than in Model 1, and come closer to “real” reversed cycles for low predator mortality (Figure 5, right column).

### 3.4 Impact of other parameters

In addition to the effectiveness of defense and predator mortality rates, other parameters may affect the phase lags; these effects are generally stronger in Model 2 than in Model 1 (Supporting information: Appendix A: Figure A3–A9). Increasing the costliness of offense *c*_*P*_, the prey carrying capacity *K* or the prey intrinsic growth rate *r* all makes the predator–prey phase lag even more pronounced in Model 2 (Supporting information: Appendix A: Figure A4, A6, A8); in Model 1, the only parameter that has a notable effect is the carrying capacity (Supporting information: Appendix A: Figure A5), while the costliness of offense and the intrinsic growth rate have little impact (Supporting information: Appendix A: Figurea A3, A7).

The only parameter that can have a major impact on the predator–prey phase lag is the speed of adaptation in the predator *G*_*P*_ (Supporting information: Appendix A: Figure A9). Rapid predator adaptation causes the synchronization between ecological and evolutionary dynamics to change, resulting in a sharp shift from reversed cycles to “regular” ¼‐lag predator–prey cycles (see van Velzen & Gaedke, 2017 for a detailed explanation of this pattern). The speed of adaptation in the prey has a comparatively minor impact on the phase lags: Faster prey adaptation tends to make defense more effective and thus results in a slightly shorter predator–prey phase lag (but still longer than antiphase) (Supporting information: Appendix A: Figure A9).

## 4 DISCUSSION

### 4.1 Using the effective prey biomass to characterize and predict predator–prey dynamics

**Figure 6:**
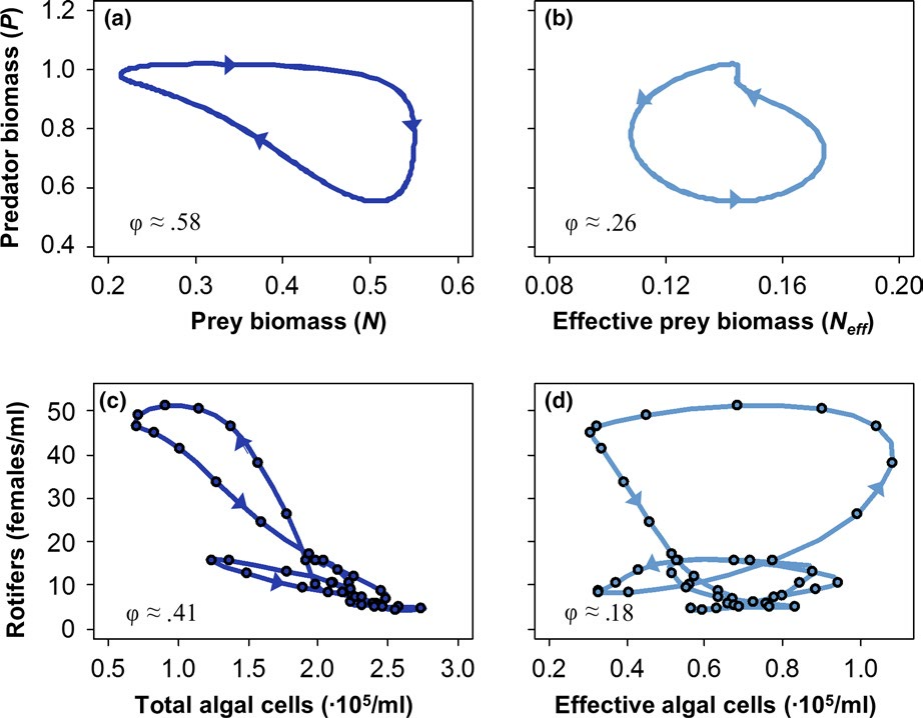
Phase space diagrams of the predator–prey dynamics shown in Figure 4a (a,b**)** and of chemostat data from Becks et al. (2010, 2012) (c,d). (a) predator versus actual prey biomass, showing reversed (clockwise) predator–prey cycles; note the elongated shape, indicating that the predator–prey phase lag is quite close to antiphase (Hiltunen et al., 2014). (b) phase plot of predator versus effective prey biomass corresponding to the dynamics in (a), showing a classic counterclockwise cycle. (c) rotifer versus total algal biomass chemostat data (cf. Figure 5a in Becks et al., 2010): The strongly elongated shape indicates a predator–prey lag close to antiphase. Note also that the direction of rotation is here still counterclockwise, corresponding to the lag slightly shorter than antiphase (φ* *≈ 0.41). (d) phase plot of predator versus effective prey biomass corresponding to the dynamics in (c), showing a counterclockwise cycle with a phase lag close to a classic ¼‐lag. For details on the analysis of the chemostat data, see Supporting information: Appendix B

The “classic” ¼‐lag cycle is a fundamental feature of predator–prey oscillations in simple predator–prey models: when plotting the dynamics on the predator–prey phase plane, the dynamics describe a counterclockwise cycle (Rosenzweig & MacArthur, 1963), where prey and predator maxima are a quarter of the cycle apart. Empirical and theoretical research has shown that evolution in prey (Becks et al., 2010; Cortez, 2011; Cortez & Ellner, 2010; Yoshida et al., 2003), predators (Cortez & Ellner, 2010; Cortez & Patel, 2017), or both (Cortez, 2015; Cortez & Weitz, 2014; Frickel et al., 2016; Mougi, 2012; Mougi & Iwasa, 2011) can give rise to predator–prey cycles with a longer phase lag. When plotting such evolution‐ or coevolution‐driven cycles on the predator–prey phase plane, the cycle shows a different shape or cycle direction. For example, antiphase cycles characteristically have an elongated and negatively correlated shape, rather than circular (Hiltunen et al., 2014; Figure 6a). When the predator–prey phase lag is longer than antiphase, so that the predator peak precedes the prey peak, the cycle direction is reversed (Figure 6a), giving rise to the terms “clockwise cycles” or “reversed cycles” (Cortez & Weitz, 2014).

While the result that coevolution can change the shape and direction of predator–prey cycles is not new, the inherent complexity and analytical intractability of coevolutionary predator–prey dynamics have impeded the development of a general and comprehensive theory. In this study, we take a new step toward a more general theory by introducing the *effective prey biomass*: The prey biomass that can be captured and assimilated by the predator. Our results show that predator dynamics in a coevolutionary predator–prey model are regulated by the effective prey biomass in the same way that they appear to be regulated by the actual prey biomass in a nonevolutionary model. Thus, even though evolution or coevolution alters the shape and direction of the predator–prey dynamics on the predator–prey phase plane, the phase diagram of predator and *effective prey* should continue to show the classic counterclockwise cycle with a ¼‐lag between predator and effective prey. The phase plots of the simulated dynamics of this study (Figure 6a,b) clearly show these patterns, as do phase plots of the algae‐rotifer chemostat experiments of Becks et al. (2010, 2012) (Figure 6c,d). We propose that this is a fundamental feature of coevolutionary predator–prey cycles, directly equivalent to the counterclockwise predator–prey cycle in nonevolutionary models. Thus, predator dynamics are expected to follow those of the effective prey with a lag of a quarter of the cycle period, just as a purely ecological predator–prey system is expected to yield this ¼‐lag cycle; although in both cases, this result is only exact when the shape of the predator–prey cycle when plotted in phase space is exactly a circle. Any distortion in the shape of the cycle (see Figure 6b) can cause slight deviations in the actual phase lag between predator and effective prey (Figure 5a, left panel). In addition, any factors that have been shown to affect the predator–prey phase lag in a nonevolutionary model, such as stage structure in the prey (Abrams & Quince, 2005), should be expected to affect the phase lag between predator and effective prey in an evolutionary model in the same way.

Under our framework, the relationship between the dynamics of effective and actual prey biomass determines the predator–prey phase relationship. This critical insight can be used both to understand what drives the shape of predator–prey cycles and to generate testable predictions. The necessary requirement for antiphase cycles, for example, is that the peak in effective prey biomass must be delayed with respect to the peak in actual prey biomass by a quarter of the cycle period. In an experimental setup with prey evolution driven by two prey clones (defended and undefended; see, e.g., Yoshida et al., 2007; Becks et al., 2010; Kasada, Yamamichi, & Yoshida, 2014), this requires that the first part of the prey peak is generated by an increase in the defended prey clone. This has indeed been shown mathematically (Jones & Ellner, 2007) and by models simulating such a scenario (Jones & Ellner, 2007; Becks et al., 2012; Supporting information: Appendix B: Figure B1a). It can also be shown with empirical data on ecoevolutionary predator–prey cycles (Becks et al., 2012; Supporting information: Appendix B: Figure B1b). Prey then become more edible in the second half of their peak (Supporting information: Appendix B: Figure B1); the peak in effective prey biomass is consequently delayed by approximately a quarter, resulting in antiphase cycles (Yoshida et al., 2003; Jones & Ellner, 2007; Becks et al., 2010; Supporting information: Appendix B: Figure B1). A similar prediction can be made for models studying continuous evolution of quantitative traits: Antiphase cycles require a temporal association between an increase in prey biomass and an increase in defense, so that prey initially become less edible and/or the predator–prey conversion efficiency decreases as the prey become more abundant. This is again reflected in the antiphase dynamics of such models (Cortez, 2011; Mougi, 2012; Mougi & Iwasa, 2011). In both clonal and quantitative genetics models, antiphase cycles result when prey peaks are initially driven by release from predation (Frickel et al., 2016; van Velzen & Gaedke, 2017) and are thus expected when defense is effective (Becks et al., 2010; Frickel et al., 2016; Jones & Ellner, 2007).

### 4.2 Conditions promoting reversed predator–prey cycles

In this study, we used the approach outlined above to generate both general and specific predictions for when *reversed cycles* should be found. Like antiphase cycles, reversed cycles require a delay in the peak in the effective prey biomass; in this case, the delay should be by half the cycle period, that is, actual and effective prey biomass should be in antiphase with each other.

While this condition is straightforward to derive, it is obviously highly restrictive: It requires that the effective biomass is highest while the actual prey biomass is lowest and vice versa. As this directly implies that changes in edibility and/or conversion efficiency must have a stronger impact on the effective prey biomass than changes in the actual prey biomass, we predicted that reversed cycles should be found when oscillations in actual prey biomass are small. This resulted in two specific predictions for the models we studied here: (a) Reversed cycles should predominantly occur under parameter ranges resulting in strong top‐down control; and (b) reversed cycles should occur less often under a trade‐off structure that promotes large‐amplitude oscillations in prey biomass (logistic growth with a trade‐off in the intrinsic growth rate, Model 1 in Equation (2)).

Both predictions were confirmed by our numerical simulations. Model 1 resulted in larger‐amplitude prey oscillations and thus resulted in shorter predator–prey phase lags than Model 2; and in both models, strong top‐down control resulted in longer phase lags and more strongly reversed cycles. Comparing these predictions to the reversed predator–prey dynamics shown in previous modeling studies (Cortez, 2015; Cortez & Weitz, 2014), they hold up as well: Their dynamics consistently showed small‐amplitude prey oscillations and a high predator/prey biomass ratio. Even more strikingly, reversed cycles in an experimental bacteria‐phage system were linked with very strong top‐down control, with prey (bacterial) densities two to three orders of magnitude below their carrying capacity (Wei et al., 2011). Thus, the hypothesis we started with, that strong top‐down control is one scenario under which we should expect reversed cycles, is confirmed by both our own simulations and by empirical data.

In our model, small‐amplitude prey oscillations were achieved by imposing conditions that resulted in very strong top‐down control. Because low predator mortality causes predator biomass to remain high, and ineffective defense makes the prey incapable of escaping the high predation pressure imposed on them, there are no pronounced peaks in prey biomass and oscillations become strongly dampened. The parameter ranges most likely to yield reversed cycles also have the effect that predator biomass generally far outweighs prey biomass (see Figure 4). While this seems a very restrictive condition, such predator/prey biomass ratios are possible in the microscopic plankton that are generally used to demonstrate ecoevolutionary cycles (del Giorgio & Gasol, 1995; Gaedke, Hochstadter, & Straile, 2002). On the other hand, such biomass ratios are generally not observed in chemostat experiments on, for example, algae‐rotifer ecoevolutionary dynamics, as the dilution rate (and, thus, predator mortality) in these experiments are typically high to very high (e.g., 0.57–1.00 per day in Yoshida et al., 2003; 0.8–0.98 per day in Yoshida et al., 2007; 0.3 per day in Becks et al., 2010). Thus, the rarity of reversed predator–prey cycles in experimental data may reflect the fact that typical experimental conditions preclude them, not that they are unlikely to occur. One way of experimentally testing our hypothesis is studying the effect of reducing the dilution rate in, for example, rotifer‐algae chemostat systems.

Strong top‐down control is not the only way to limit the amplitude of prey oscillations, however. Another way to achieve this is for the prey to remain close to the carrying capacity at all times (see model dynamics in Yoshida et al., 2007)—that is, when top‐down control is very weak, rather than very strong. We do not see this dynamic in our model, but other models might be constructed in which reversed cycles can be demonstrated under these conditions.

Previous models for reversed predator–prey cycles pointed toward a critical role for the costs of offense (Cortez, 2015; Cortez & Weitz, 2014). We did confirm that a higher costliness of offense results in longer phase lags in Model 2, but found no real effect in Model 1 (Supporting information: Appendix A: Figures A3, A4). The mechanism responsible for the longer phase lags in Model 2 is essentially the same as the one proposed by Cortez and Weitz (2014): The relative importance of the conversion efficiency (compared to edibility) on the peak in effective prey biomass increases with the costliness. As the conversion efficiency typically lags behind edibility (see Figures 3 and 4), this results in a more pronounced delay in peak effective prey. However, that we do not find this result in Model 1, where prey biomass oscillations are far more pronounced, underscores that a high costliness for offense is not sufficient to produce reversed cycles: it still requires small‐amplitude prey oscillations.

The absence of large‐amplitude oscillations in actual prey biomass is thus the strongest, most consistently confirmed requirement for reversed cycles. It is therefore striking that this prediction was entirely missing from previous models, even though their results appear to confirm it (Cortez, 2015; Cortez & Weitz, 2014). This difference in model predictions directly reflects the strong differences in the approaches used to derive them. The approach of fast‐slow dynamics (Cortez, 2015; Cortez & Ellner, 2010) separates evolutionary and ecological timescales, with evolutionary changes being much faster than ecological changes. In addition, disruptive selection was assumed in the models studying reversed cycles (Cortez, 2015; Cortez & Weitz, 2014), which, when combined with very rapid evolution, resulted in rapid shifts between, for example, extremely edible and extremely inedible prey. This combination of slow ecological dynamics and rapid, high‐impact shifts in predator and prey traits results in a highly artificial dynamic where trait changes are always the main determinant of the effective prey biomass. The condition we derived as necessary for reversed cycles—that trait changes must be decisive for the dynamics of the effective prey biomass—is then always met, but this is an artifact of the modeling approach. However, no empirical evidence exists that evolutionary changes are much faster than ecological changes (DeLong et al., 2016); under more realistic scenarios where ecological and evolutionary changes occur on similar timescales or where evolutionary changes are slower (DeLong et al., 2016; Ellner et al., 2011), changes in actual prey biomass can leave a very strong imprint on the dynamics of the effective prey biomass, as can be clearly seen in our results. Our model predictions are thus more realistic and more likely to be generally applicable to real predator–prey systems.

## 5 CONCLUSIONS

The complexity of coevolutionary predator–prey models typically makes mechanistic insights difficult, if not impossible, to extract. Attempts to achieve such mechanistic insights often rely on separating ecological and evolutionary timescales, simplifying the system to make it more analytically tractable. In this study, we demonstrate the importance of considering ecoevolutionary feedbacks in all their complexity: This allowed us to make new predictions for the conditions promoting reversed cycles that could not have been derived from approaches using separate timescales. Moreover, while coevolutionary predator–prey dynamics are especially unwieldy due to the multitude of feedbacks, we show that fundamental understanding can still be gained even without mathematical tools for simplifying these dynamics. The critical insight of our new approach, which predator dynamics are regulated by the dynamics of the effective prey biomass, is an important step toward a comprehensive theory on ecoevolutionary predator–prey dynamics.

## CONFLICT OF INTEREST

None declared.

## AUTHOR CONTRIBUTIONS

UG and EV developed the idea for this study. EV developed the model and analysis, ran the simulations and analyzed the results, and drafted the manuscript. UG contributed substantially to the editing and revising of the manuscript.
