# Supplementary material for: Reversed predator–prey cycles are driven by the amplitude of prey oscillations
Source: Ecol Evol. 2018 May 24;8(12):6317–29. doi: 10.1002/ece3.4184 (PMC6024131; doi:10.1002/ece3.4184)
Supplement: Supplementary file 1 [file ECE3-8-6317-s001.pdf]

Supplementary Material belonging to

Ellen van Velzen, Ursula Gaedke, *Reversed predator-prey cycles are driven by the amplitude of prey oscillations*

Contents

Appendix A: Supporting figures 2
Appendix B: effective prey biomass in systems with two discrete prey types 11
References 15

Appendix A: Supporting figures

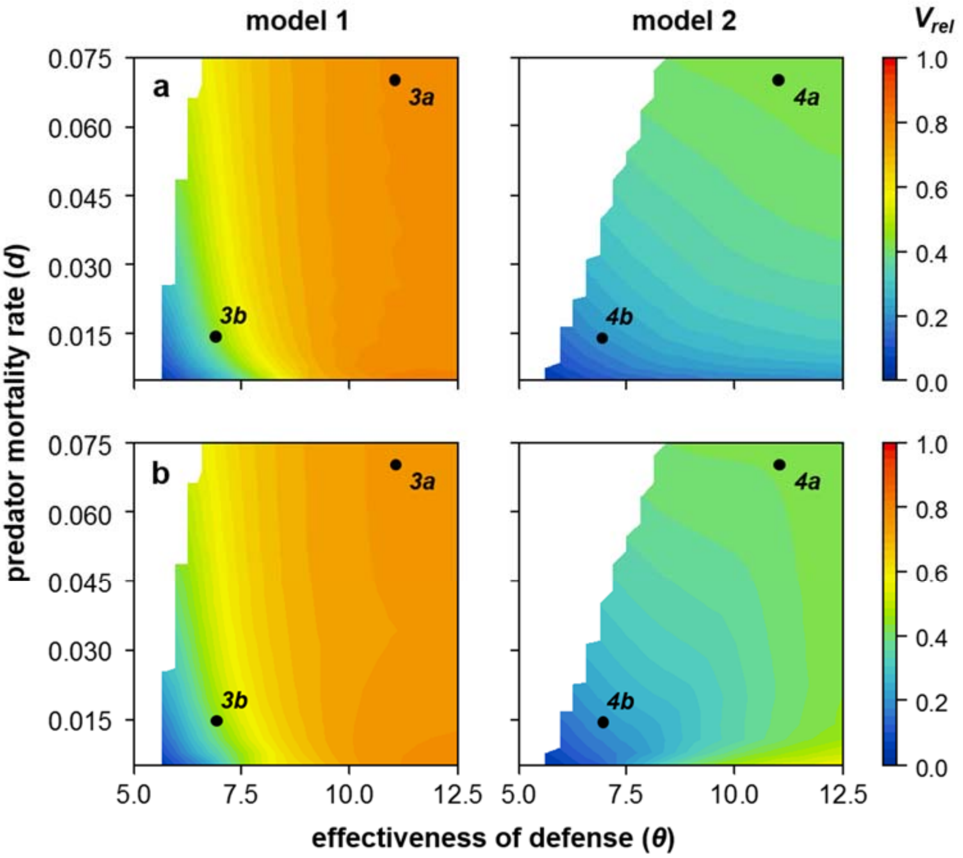

Figure A1: The relative variability in prey biomass, using two different measures of variability. **a**: a non-parametric measure of variability (the difference between the 10% and 90% quantiles; Kenitz et al. 2017). **b**: amplitudes (the difference between maximum and minimum values of the timeseries). All parameter values are the same as in Fig. 2 in the main text. White regions indicate stable equilibria.

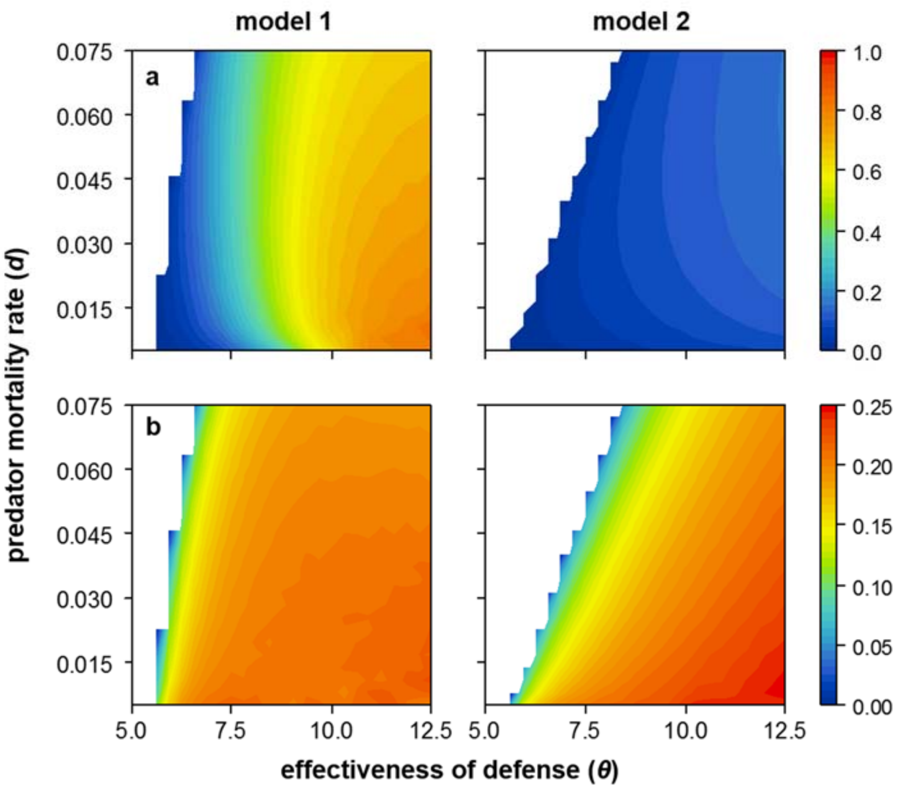

Figure A2: Standard deviations in the dynamics of **(a)** prey biomass and **(b)** the product of edibility and conversion efficiency for the two models, as a function of predator mortality d and the effectiveness of defense θ. All parameter values are the same as in Fig. 2-5 in main text (see Table 1). Colours denote the standard deviations; note that different scales are used for **a** and **b**, but the same scale is used for both models to allow direct comparison. White regions indicate stable equilibria.

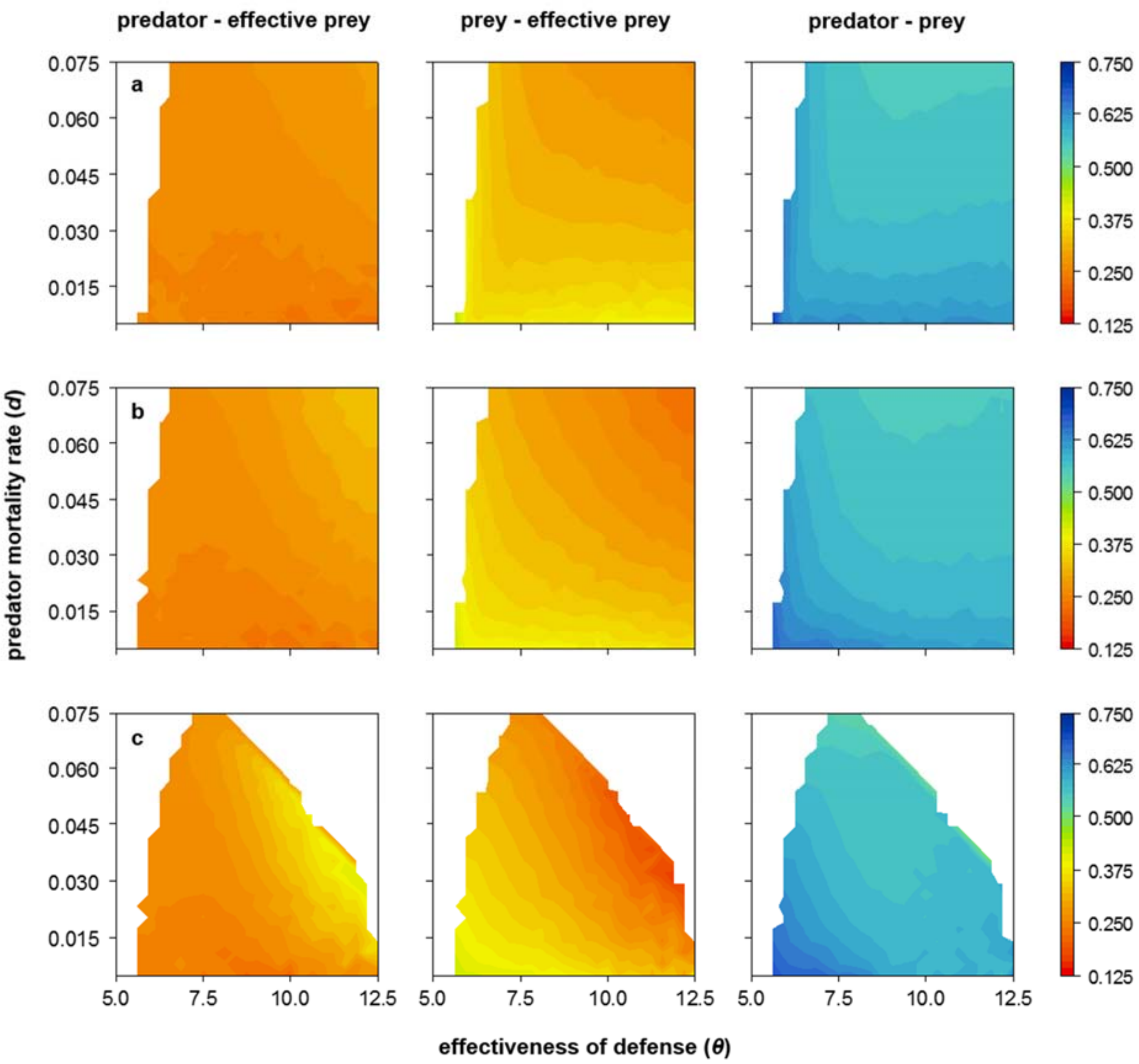

Figure A3: Phase lags between predator biomass and effective prey biomass (left), between effective and actual prey biomass (middle) and between predator and actual prey biomass (right) for model 1. Colours denote the phase lag, as given in the legend on the right; white regions indicate stable equilibria. **a**: $c_P = 3.0$; **b**: $c_P = 3.5$; **c**: $c_P = 4.0$. Other parameter values are given in Table 1 in main text.

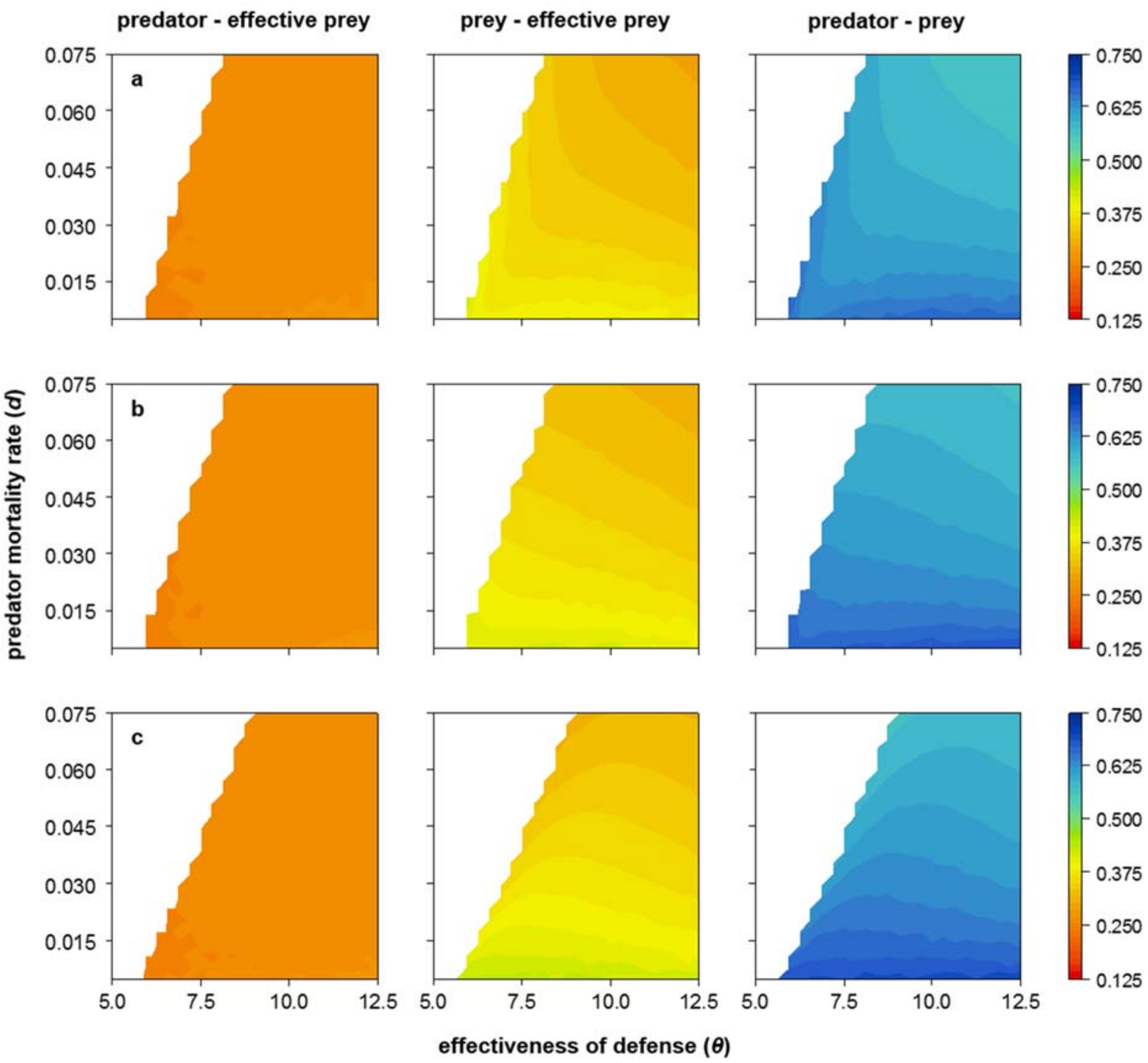

Figure A4: Phase lags between predator biomass and effective prey biomass (left), between effective and actual prey biomass (middle) and between predator and actual prey biomass (right) for model 2. Colours denote the phase lag, as given in the legend on the right; white regions indicate stable equilibria. **a**: c_P = 3.0; **b**: c_P = 3.5; **c**: c_P = 4.0. Other parameter values are given in Table 1 in main text.

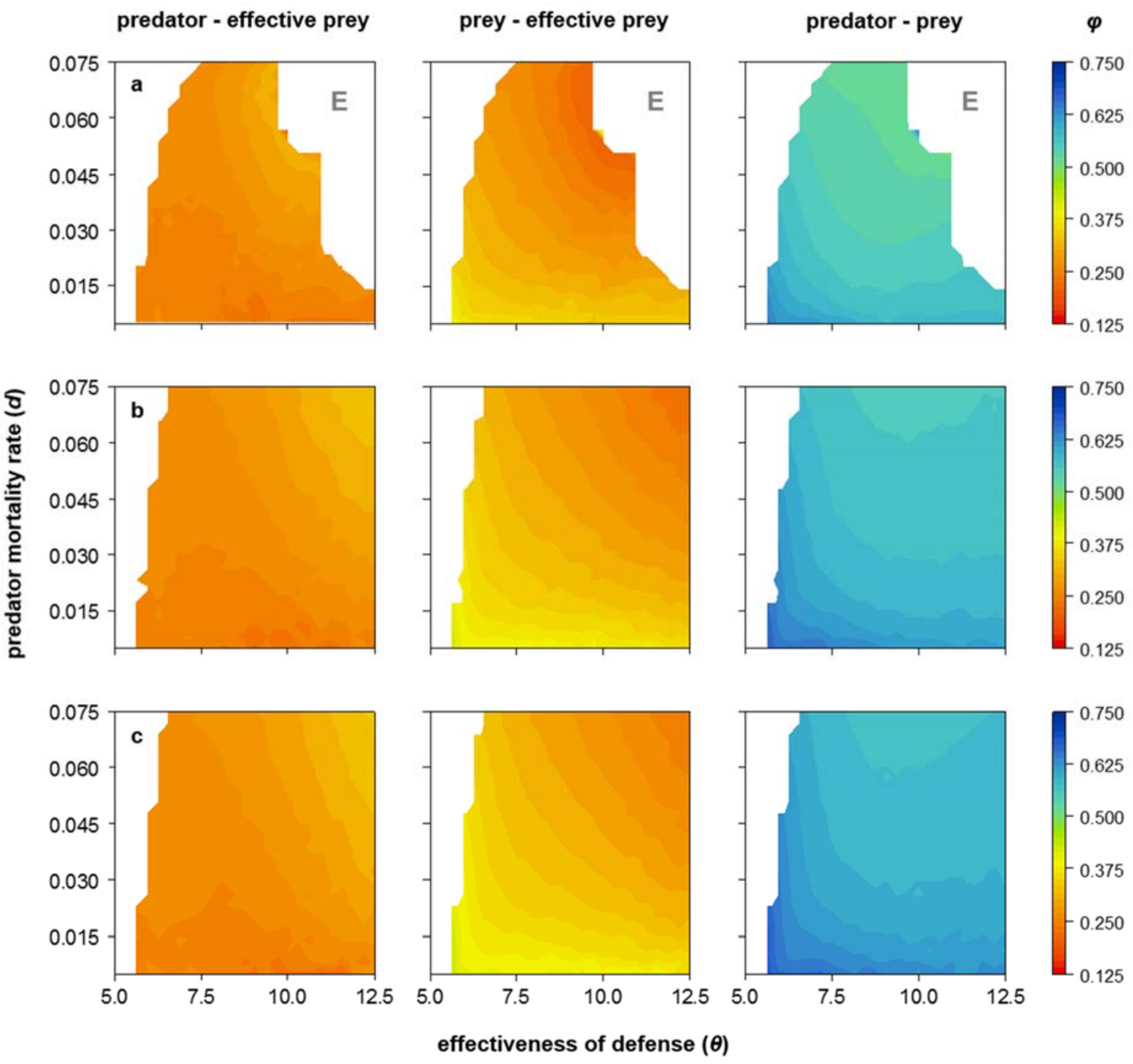

Figure A5: Phase lags between predator biomass and effective prey biomass (left), between effective and actual prey biomass (middle) and between predator and actual prey biomass (right) for model 1, for three different values for the carrying capacity K. Colours denote the phase lag, as given in the legend on the right; white regions indicate stable equilibria. The regions marked "E" in (**a**) indicate extinction of the predators. **a**: $K = 1$; **b**: $K = 2$; **c**: $K = 4$. Other parameter values are given in Table 1 in main text.

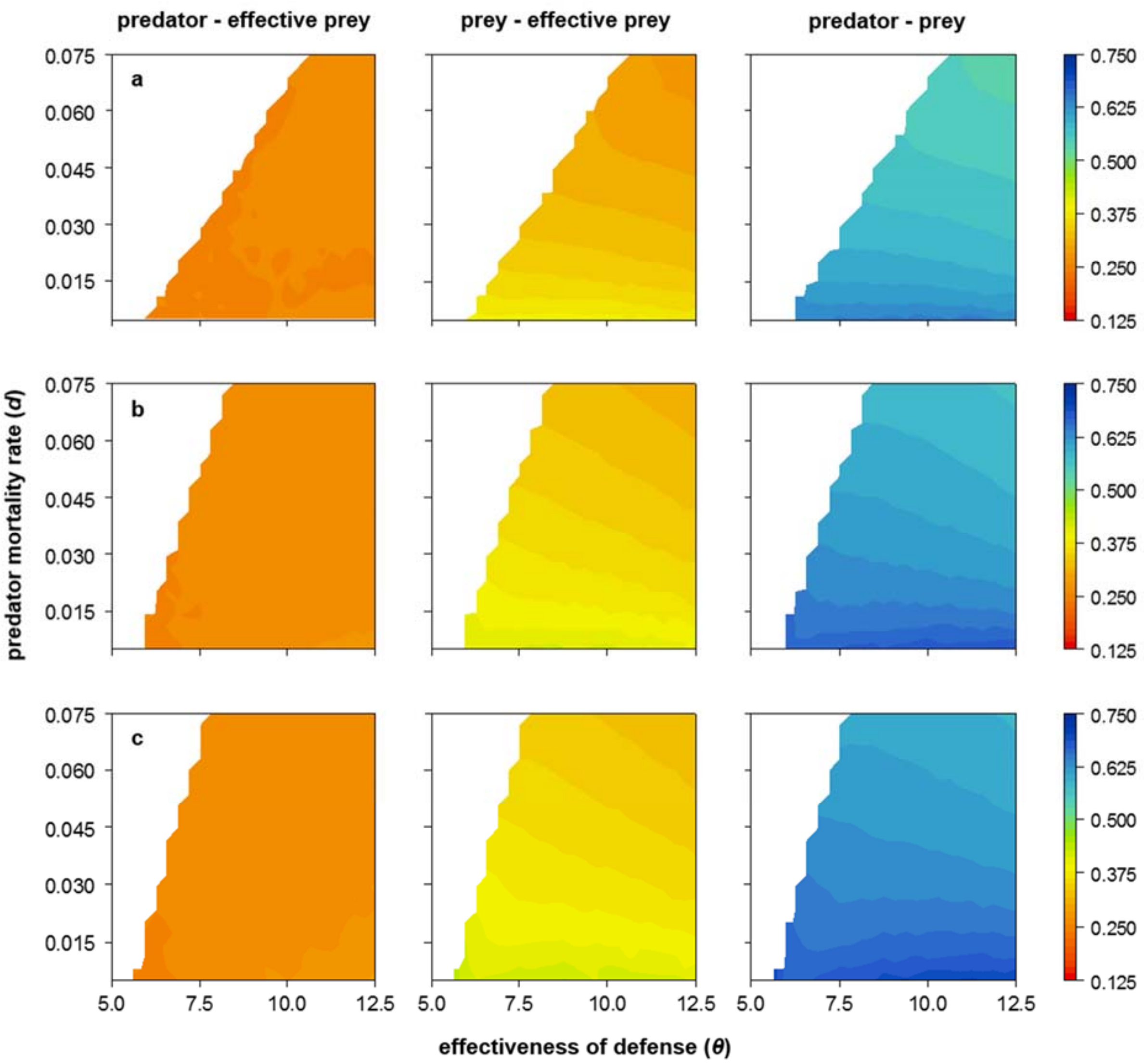

Figure A6: Phase lags between predator biomass and effective prey biomass (left), between effective and actual prey biomass (middle) and between predator and actual prey biomass (right) for model 2, for three different values for K_r. Colours denote the phase lag, as given in the legend on the right; white regions indicate stable equilibria. **a**: $K_r = 1$; **b**: $K_r = 2$; **c**: $K_r = 4$. Other parameter values are given in Table 1 in main text.

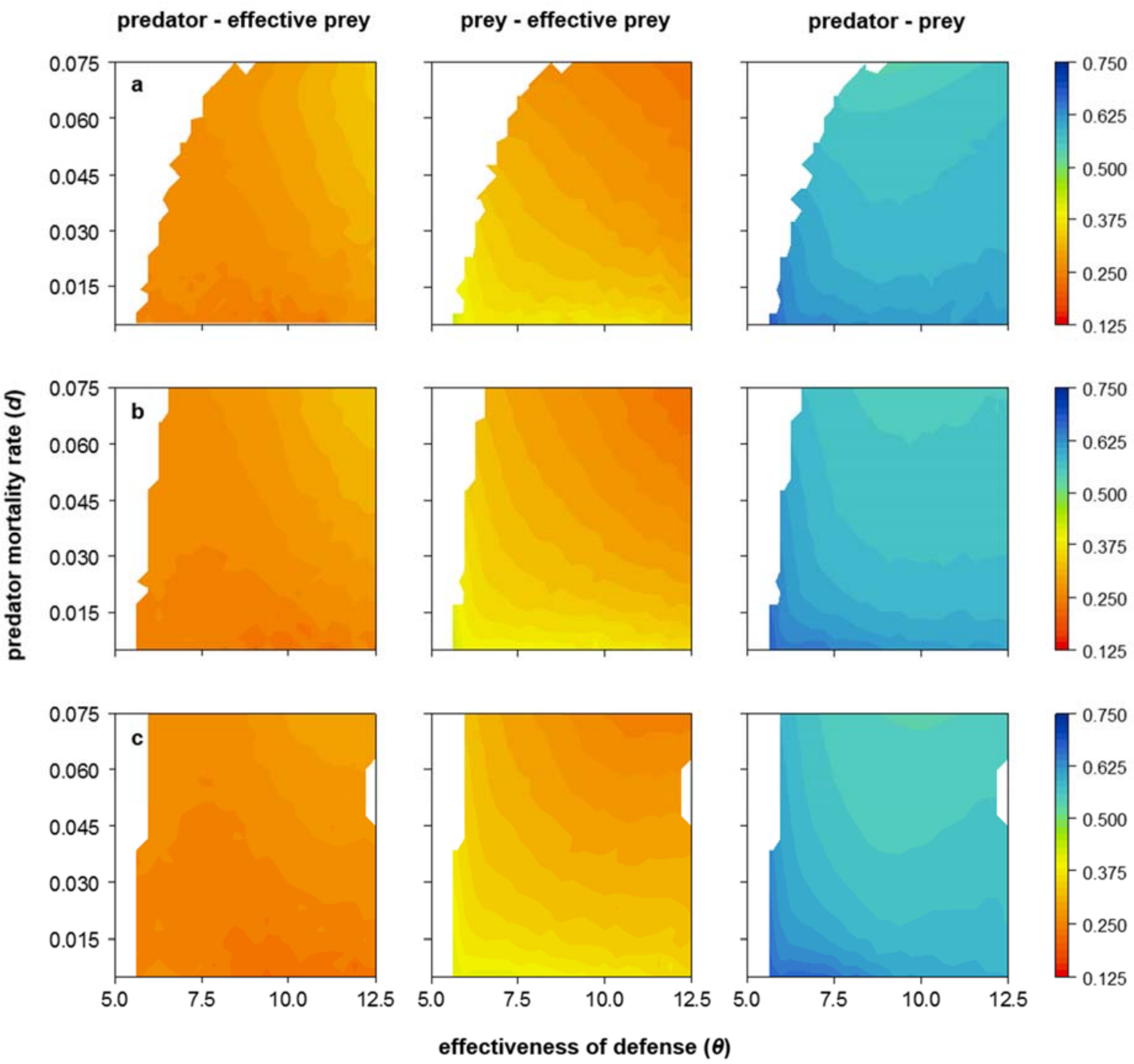

Figure A7: Phase lags between predator biomass and effective prey biomass (left), between effective and actual prey biomass (middle) and between predator and actual prey biomass (right) for model 1, for three different values for the intrinsic growth rate *r*. Colours denote the phase lag, as given in the legend on the right; white regions indicate stable equilibria. **a**: $r = 0.5$; **b**: $r = 1$; **c**: $r = 2$. Other parameter values are given in Table 1 in main text.

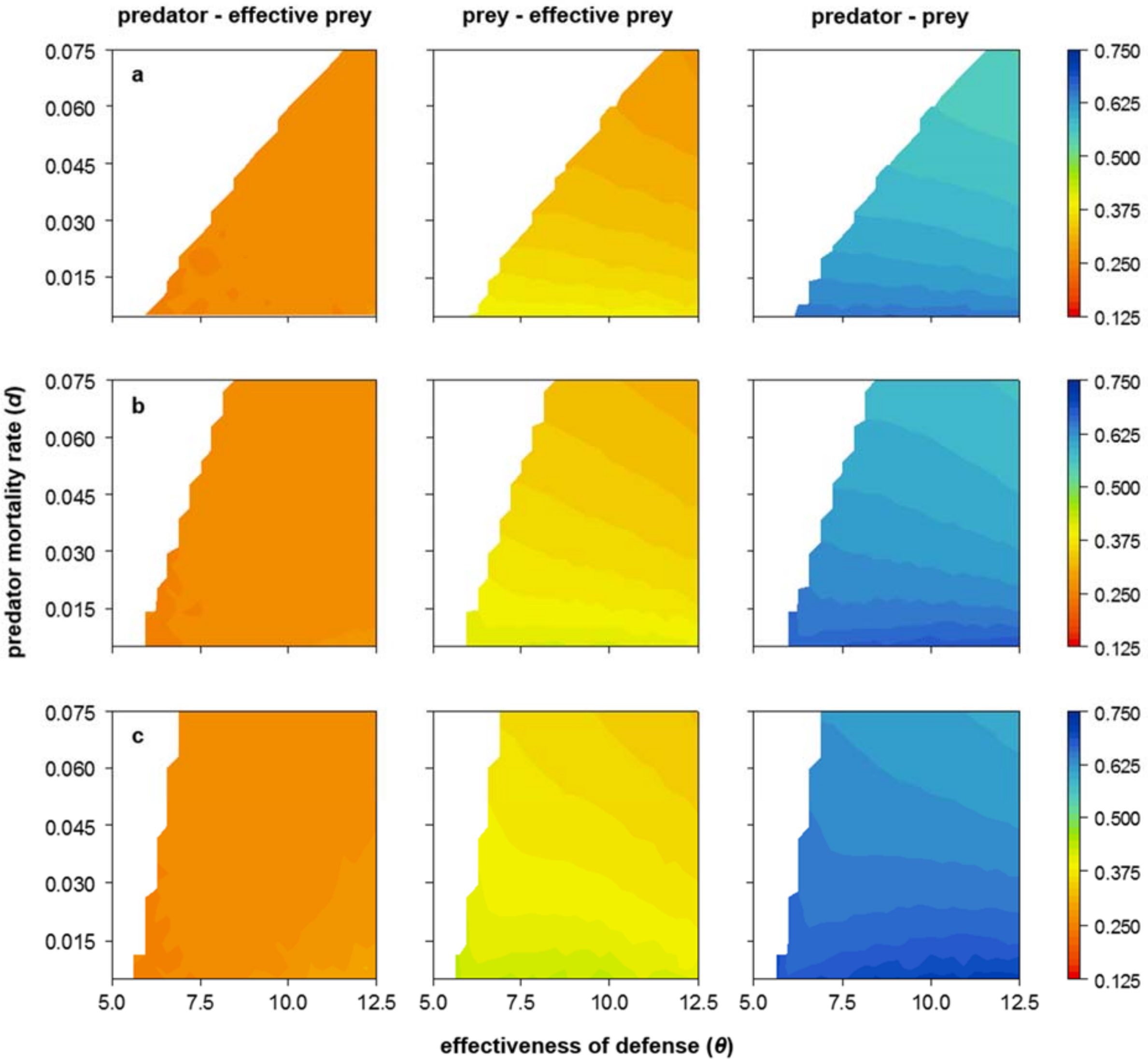

Figure A8: Phase lags between predator biomass and effective prey biomass (left), between effective and actual prey biomass (middle) and between predator and actual prey biomass (right) for model 2, for three different values for the intrinsic growth rate r. Colours denote the phase lag, as given in the legend on the right; white regions indicate stable equilibria. **a**: $r = 0.5$; **b**: $r = 1$; **c**: $r = 2$. Other parameter values are given in Table 1 in main text.

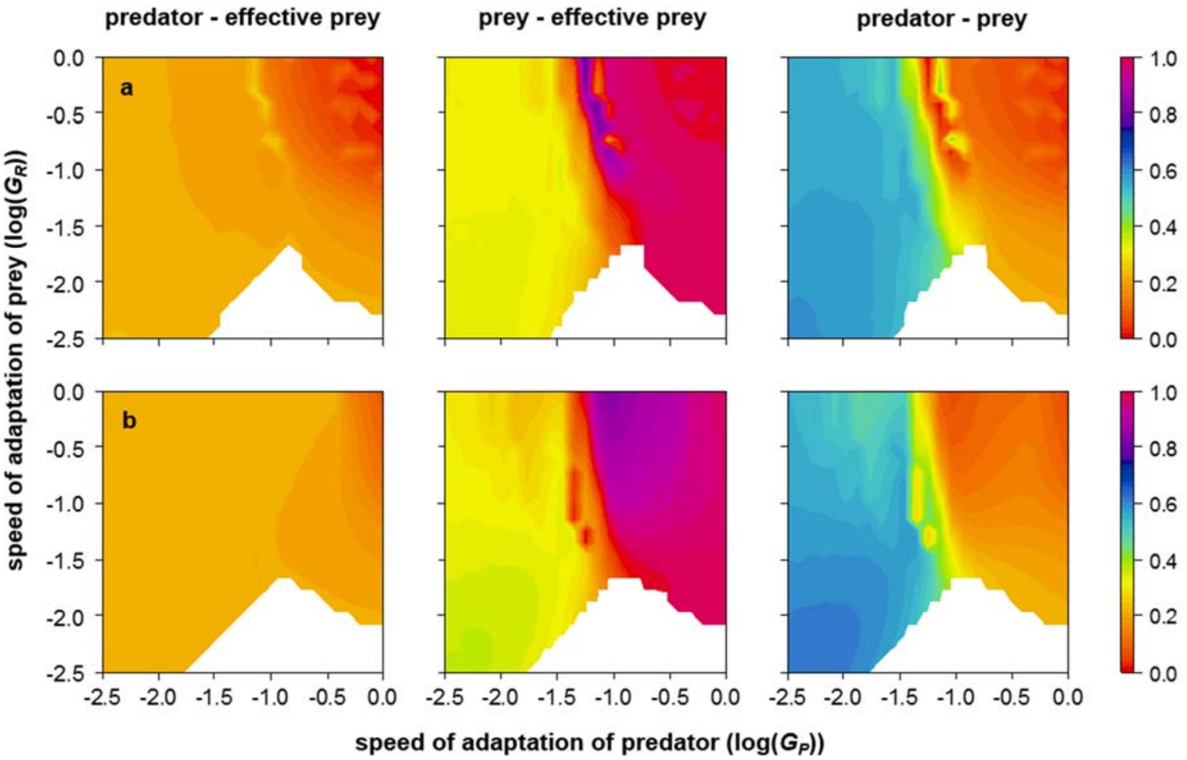

Figure A9: Phase lags between predator biomass and effective prey biomass (left), between effective and actual prey biomass (middle) and between predator and actual prey biomass (right) depending on the speed of adaptation of the prey (G_R) and predator (G_P). **a**: model 1; **b**: model 2. In both, $d = 0.015$, $\theta = 7$ (cf. Fig. 2-4 in the main text); other parameter values are given in Table 1 in the main text. Colours denote the phase lag, as given in the legend on the right. Note that the colour scale is slightly different from Fig. 4 and Figs. A3-A8, to accommodate the in-phase dynamics between actual and effective prey biomass when G_P is high (see van Velzen & Gaedke 2017 for a detailed explanation of this pattern).

Appendix B: effective prey biomass in systems with two discrete prey types

In this appendix, we show in more detail how the concept of the effective prey biomass can be applied to systems where prey can be divided into discrete phenotypes (e.g. an undefended and a defended type), rather than a single prey with a continuous defense trait. We apply the same analysis both to a simulated time series of a simple predator prey model with two prey clones (section 1 below), and to an experimental dataset with genetic variation in defense (section 2).

B1. A two-phenotype predator-prey model

Rather than a single prey with a continuously changing degree of defense, we consider a prey population consisting of two discrete prey phenotypes or clones, one of which is highly edible ("undefended") while the other has a lower edibility ("defended"). We assume prey growth with self-limitation, and a Holling type II functional response for the predator (cf. model 2 in the main Methods):

$$\begin{aligned}
\frac{dN_1}{dt} &= N_1\left(r_1 - \frac{N_1+N_2}{K} - \frac{ap_1P}{1+ah\left(N_1+pN_2\right)}\right)\\
\frac{dN_2}{dt} &= N_2\left(r_2 - \frac{N_1+N_2}{K} - \frac{ap_2P}{1+ah\left(N_1+pN_2\right)}\right)\\
\frac{dP}{dt} &= P\left(\frac{\varepsilon a\left(p_1R_1+p_2R_2\right)}{1+ah\left(p_1R_1+p_2R_2\right)} - d\right)
\end{aligned} \qquad \text{(B1)}$$

where N_1 and N_2 are the biomasses of the undefended and defended prey clones, respectively, and P is the predator biomass (we assume no adaptation is taking place in the predator). r_1 and r_2 are the intrinsic growth rates of the two prey clones; K is their shared carrying capacity; a and h are the maximum attack rate and the handling time of the predator, and ε is the conversion efficiency of captured prey into predator biomass. Finally, p_1 and p_2 represent the edibilities of the two prey clones, resulting in the realized attack rates $a{\cdot}p_1$ and $a{\cdot}p_2$ on N_1 and N_2, respectively.

In the following, we assume that prey N_1 is completely undefended ($p_1 = 1$). Further, we assume a trade-off between defense and growth: N_2 is less edible ($p_2 < 1$) but has a lower intrinsic growth rate ($r_2 < r_1$).

Defining mean edibility $\bar{p}$ and effective prey biomass N_{eff}

If N_2 is completely inedible ($p_2 = 0$), the effective prey biomass is exactly equal to the biomass of the undefended prey N_1. If N_2 is still partly edible ($p_2 > 0$), the effective prey biomass is given by

$$N_{eff} = N_1 + p_2N_2. \qquad \text{(B2)}$$

The mean edibility of the prey is defined as

$$\overline{p} = \frac{N_1 + p_2 N_2}{N_1 + N_2}. \tag{B3}$$

If $p_2 = 0$, this reduces to the fraction of N_1 in the total prey biomass. Since the numerator in eq. (B3) is the expression for the effective prey biomass (see eq. (B2)), it follows that the effective prey biomass is the product of the total prey biomass and the average prey edibility:

$$N_{eff} = \overline{p}\left(N_1 + N_2\right). \tag{B4}$$

Data analysis

Following the Methods in the main text, we calculated three phase lags φ: between predator and total ("actual") prey biomass, between predator and effective prey biomass, and between effective and total prey biomass. Phase lags are expressed as $0 \leq \varphi \leq 1$, where 0 and 1 indicate in-phase cycles (no lag). $\varphi \approx 0.25$ indicates classic ¼-lag cycles and $\varphi \approx 0.5$ indicates antiphase cycles.

B2. Applied to experimental data: chemostat experiments of Becks et al. (2010, 2012)

Second, we applied the same approach to data of chemostat experiments, in which the predator-prey cycles followed an antiphase dynamic (data from Becks et al. 2010, 2012). These experiments were conducted with two clones of the green alga *Chlamydomonas reinhardtii* as prey, and the rotifer *Brachionus calyciflorus* as the predator. One algal clone is undefended, while the other has a heritable defense against predation in the form of colony formation: cells in larger clumps (≥ 8 cells, Becks et al. 2012) cannot be ingested by the rotifers. This defense comes at the cost of a reduced growth rate (Becks et al. 2010). In chemostat runs with both clones present, antiphase predator-prey cycles were observed, with changes in mean clump size on the same timescale as the predator-prey cycle ("eco-evolutionary cycles"; Becks et al. 2010). Here we illustrate how these dynamics can be explained by the dynamics of the *effective prey biomass* in the first of these four chemostat runs (cf. Fig. 5a in Becks et al. 2010; Fig. 1a in Becks et al. 2012).

Defining edible and inedible prey, mean edibility, and effective prey biomass

Is has previously been shown prey clumps smaller than 8 cells are edible, while larger clumps are inedible (Becks et al. 2012). We therefore assigned all prey with a clump size < 8 an edibility of 1 ($p_1 = 1$, "undefended"), and all prey with a larger clump size an edibility of 0 ($p_2 = 0$, "defended"). The mean edibility was calculated as the fraction of the total prey biomass that was undefended (cf. Eq. (B3)). Finally, the effective prey biomass was calculated by multiplying the total prey biomass with the mean edibility (cf. Eq. (B4)). As far as we are aware, clump size plays no role in the conversion efficiency of algae to predators in this system.

Data analysis

Analysis of the data was carried out in R version 3.1.2. Data were smoothed for analysis using local cubic regression. The data of the first 10 days were excluded for calculating the phase lags, as the system is expected to exhibit transient dynamics.

To determine the phase lags φ in fractions of the period length of the oscillations, the period length t_{per} was determined using the autocorrelation in the prey dynamics, and the lags λ_{ij} (in days) between total prey, effective prey and predator were determined using the cross-correlation function. The phase lags were then calculated as $\varphi = \lambda / t_{per}$.

Results

The dynamics of the simulations and the experimental chemostat data show highly similar patterns (Fig. B1). The initial increase in total prey biomass is driven by an increase in the defended prey (Fig. B1, top panels); as the prey now become highly inedible (middle panels), predator biomass declines to low numbers (bottom panels), in turn allowing the undefended prey to establish. Thus, the peak in the undefended prey falls together with the last part of the peak in total prey (top panels). This causes the dynamics of the effective prey biomass to deviate from those of the total prey biomass: the effective prey biomass remains low during the first half of the peak in total prey biomass, resulting in peaks in effective prey biomass that are delayed with respect to the peaks in total prey biomass (middle and bottom panels).

Phase lags. In both simulated and experimental data, the phase lag between predator and total prey biomass is close to antiphase ($\varphi \approx 0.44$ in simulation, $\varphi \approx 0.41$ in experiment), and the phase lag between predator and effective prey biomass close to a ¼-lag ($\varphi \approx 0.23$ and $\varphi \approx 0.18$ in simulation and experiment, respectively). Thus, the longer lag between predator and total prey biomass can be explained by the fact that the dynamics of the effective prey biomass are delayed with respect to those of the total prey biomass ($\varphi \approx 0.21$ and $\varphi \approx 0.26$ in simulation and experiment, respectively).

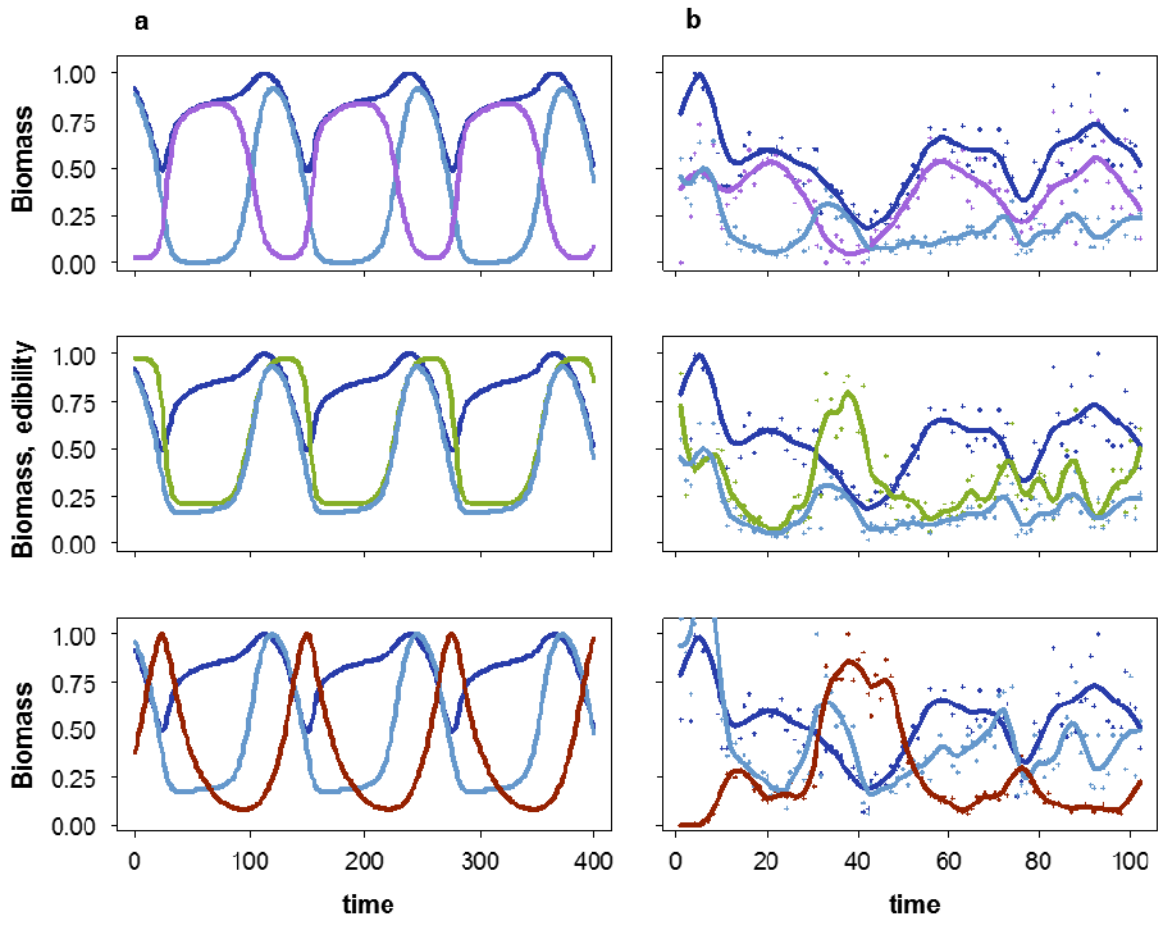

Figure B1: Dynamics of **a** the model in eqs. (B1-B3); **b** one of the chemostat runs in Becks et al. 2010, 2012; symbols represent daily measurement data, and lines the smoothed fits. Top panels: dynamics of total prey biomass (dark blue), undefended prey (light blue) and defended prey (purple). Middle panels: dynamics of total prey (dark blue), mean edibility (green) and effective prey (light blue). Bottom panels: dynamics of total prey (dark blue), effective prey (light blue) and predator (red) biomass. Both model and experimental data were normalized to the highest prey biomass (for prey, defended prey, undefended prey, and effective prey) or the highest predator biomass (for the predator), so that all values are between 0 and 1. Parameters in **a**: $r_1 = 1.0$, $r_2 = 0.8$, $K = 1$, $a = 1.5$, $h = 1$, $p_2 = 0.2$, $\varepsilon = 0.3$, $d = 0.1$.

References

Becks, L., S. P. Ellner, L. E. Jones, and N. G. Hairston Jr. 2010. Reduction of adaptive genetic diversity radically alters eco-evolutionary community dynamics. Ecology Letters 13:989-997.

Becks, L., S. P. Ellner, L. E. Jones, and N. G. Hairston Jr. 2012. The functional genomics of an eco-evolutionary feedback loop: linking gene expression, trait evolution, and community dynamics. Ecology Letters 15:492-501.

Kenitz, K. M., A. W. Visser, P. Mariani, and K. H. Andersen. 2017. Seasonal succession in zooplankton feeding traits reveals trophic trait coupling. Limnology and Oceanography 62:1184-1197.

van Velzen, E., and U. Gaedke. 2017. Disentangling eco-evolutionary dynamics of predator-prey coevolution: the case of antiphase cycles. Scientific reports 7:17125-017-17019-4.
